# Critical care outcomes in patients with pre-existing pulmonary hypertension

**DOI:** 10.1186/2197-425X-3-S1-A358

**Published:** 2015-10-01

**Authors:** KB Bauchmuller, R Condliffe, C Billings, Y Arunan, GH Mills

**Affiliations:** Sheffield Teaching Hospitals NHS Foundation Trust, Anaesthesia and Critical Care, Sheffield, United Kingdom; National Pulmonary Hypertension Service, Pulmonary Vascular Disease Unit, Royal Hallamshire Hospital, Sheffield Teaching Hospitals NHS Foundation Trust, Sheffield, United Kingdom

## Introduction

Pulmonary hypertension (PH) has traditionally been associated with a poor prognosis. However, recent advances in the understanding of its pathophysiology have opened new avenues for treatment [1]. Of note, there are few data on outcome of patients with pre-existing PH managed in critical care.

## Objectives

To assess critical care outcomes in patients with pre-existing pulmonary hypertension and evaluate potential predictors of survival.

## Methods

We conducted a retrospective observational study of patients with PH admitted to the critical care department (CCD) in a UK national pulmonary hypertension referral centre between April 2000 and July 2014. Critical care data on demographics, admission details, physiological and biochemical parameters as well as treatment modalities in the CCD were collected from our electronic patient record system (Metavision). These were amalgamated with data on WHO functional state, right heart catheter examinations and shuttle walk distance from the PH unit clinical database. Predictors of critical care and hospital survival were assessed using uni- and multivariate logistic regression analysis. Results are expressed as mean ± SD and odds ratios (OR) with 95% confidence intervals (CI).

## Results

One hundred and forty seven patients were included in the study, accounting for 169 individual admission episodes to critical care. Baseline demographics and PH data are shown in Table 1.

**Table 1:** Baseline characteristics

Age (years)	50.5 ± 17.2
Sex M/F (%)	34/66
PH diagnosis group: 1 (PAH), 2 (Left heart disease), 3 (Lung disease), 4 (CTEPH), 5 (Miscellaneous). (%)	64/5/6/17/7
WHO functional class: 1/2/3/4 (%)	0/13/61/26
mRAP (mmHg)	11.02 ± 6.88
mPAP (mmHg)	47.26 ± 12.82
PAWP (mmHg)	10.89 ± 4.30
CI (L.min-1.m-2)	2.81 ± 1.12
PVR (dyn.s.cm-5)	697 ± 437
ISWT distance (m)	205 ± 158

Medical, surgical and obstetric categories accounted for 79%, 14% and 8% of admissions. Hospital survival in these subgroups was 65, 91 and 85%, respectively. Thirty percent of patients received CPAP, 9% bilevel NIV, 5% invasive ventilation, 23% vasopressors, 19% inotropes and 12% CVVH. These therapeutic modalities were associated with a hospital survival of 51, 47, 11, 41, 34 and 57%, respectively. Overall, 76% of patients survived to discharge from critical care and 70% left hospital alive. Several parameters at admission to the CCD were identified as predictors of hospital discharge at univariate and multivariate logistic regression analysis, most notably Sp02/Fi02 ratio, serum lactate and serum sodium concentration (see Table 2).

**Table 2:** Admission parameters predicting hospital survival

	Univariate			Multivariate		
	OR	95% CI	p	OR	95% CI	p
Age	0.986	(0.967-1.005)	0.15			
HR	1.026	(1.009-1.044)	0.003			
MAP	0.980	(0.961-1.000)	0.052			
Sp02/Fi02	0.994	(0.990-0.997)	0.001	0.994	(0.989-0.999)	0.01
Lactate	1.655	(1.252-2.188)	< 0.001	1.644	(1.244-2.172)	< 0.001
Na+	0.920	(0.864-0.979)	0.008	0.891	(0.827-0.959)	0.002
Urea	1.047	(1.008-1.087)	0.019			
Platelets	0.996	(0.992-0.999)	0.02			

## Conclusions

Overall, more than two thirds of patients with pre-existing PH admitted to critical care survived to hospital discharge. Medical reasons for admission were associated with a worse outcome compared with surgical and obstetric indications. Independent predictors of hospital mortality were low serum sodium, high lactate and low Sp02/Fi02 ratio, reflecting heart failure, poor cardiac output and poor oxygenation.
